# Investigating the Acute Metabolic Effects of the N-Methyl Carbamate Insecticide, Methomyl, on Mouse Liver

**DOI:** 10.3390/metabo13080901

**Published:** 2023-08-01

**Authors:** Amy M. Groswald, Tyler C. Gripshover, Walter H. Watson, Banrida Wahlang, Jianzhu Luo, Loretta L. Jophlin, Matthew C. Cave

**Affiliations:** 1 Division of Gastroenterology, Hepatology, and Nutrition, Department of Medicine, School of Medicine, University of Louisville, Louisville, KY 40202, USA; gros79@osumc.edu (A.M.G.); bert.watson@louisville.edu (W.H.W.); banrida.wahlang@louisville.edu (B.W.); jianzhu.luo@louisville.edu (J.L.); loretta.jophlin@louisville.edu (L.L.J.); 2 Department of Pharmacology & Toxicology, School of Medicine, University of Louisville, Louisville, KY 40202, USA; tcgrip01@louisville.edu; 3 The Hepatobiology and Toxicology Center, University of Louisville, Louisville, KY 40202, USA; 4 The University of Louisville Alcohol Research Center, Louisville, KY 40202, USA; 5 The Center for Integrative Environmental Health Sciences, Louisville, KY 40202, USA; 6 The University of Louisville Superfund Research Center, Louisville, KY 40202, USA; 7 Department of Biochemistry & Molecular Genetics, School of Medicine, University of Louisville, Louisville, KY 40202, USA; 8 Robley Rex Veterans Affairs Medical Center, Louisville, KY 40206, USA

**Keywords:** toxicant-associated steatotic liver disease, toxicant-associated steatohepatitis, pesticide, environmental liver disease, endocrine disruptor, metabolic disrupting chemical

## Abstract

Many pesticides have been identified as endocrine and metabolism-disrupting chemicals with hepatotoxic effects. However, data are limited for insecticides in the n-methyl carbamate class, including methomyl. Here, we investigate the liver and systemic metabolic effects of methomyl in a mouse model. We hypothesize that methomyl exposure will disrupt xenobiotic and intermediary metabolism and promote hepatic steatosis in mice. Male C57BL/6 mice were exposed daily to 0–5 mg/kg methomyl for 18 days. Mice were fed water and regular chow diet ad libitum. Metabolic phenotyping was performed, and tissue samples were collected. Effects were generally greatest at the highest methomyl dose, which induced *Cyp1a2*. Methomyl decreased whole body weight while the liver:body weight and testes:body weight ratios were increased. Hepatic steatosis increased while plasma LDL decreased. Fasting blood glucose and the glucose tolerance test area under the curve decreased along with hepatic glycogen stores. Methomyl, however, did not increase liver oxidative stress or injury. Collectively, these data demonstrate that methomyl disrupts hepatic xenobiotic and intermediary metabolism while increasing the testes:body weight ratio, suggesting that it may be an endocrine disrupting chemical. Besides methomyl’s known action in cholinesterase inhibition, it may be involved in aryl hydrocarbon receptor activation. The potential impact of n-methyl carbamate insecticides on metabolic health and diseases, including toxicant-associated steatotic liver disease (TASLD), warrants further investigation.

## 1. Introduction

Methomyl (Lannate LV^®^, Nudrin LV^®^) is an n-methyl carbamate insecticide which has been used for agricultural applications since it was first registered in 1968 [1]. Like organophosphates, methomyl targets insects and other pests via inhibition of acetylcholinesterase. N-methyl carbamates are generally favored for their low vapor pressure and rapid photodegradation leading to low environmental persistence [2]; however, acute ingestion or inhalation exposure to n-methyl carbamates, including methomyl, can be extremely neurotoxic to humans, even leading to respiratory paralysis and death. Methomyl is registered for a variety of agricultural uses including field crops and tobacco. However, due to suspected toxicological health effects, methomyl was classified as a restricted use pesticide in the early 2010′s [1]. According to the US EPA, methomyl is a moderate-to-highly toxic compound under oral or inhalation exposure. Since methomyl’s observed human toxicity, government agencies and manufacturers have limited its applications.

Some insecticides are endocrine- and metabolism-disrupting chemicals (EDCs/MDCs) [3]. Given its central role in xenobiotic detoxification, the liver is a primary target of numerous ingested toxicants including pesticides such as organophosphates, which have been shown to alter energy homeostasis and lipid metabolism as well as to act as MDCs [4]. Indeed, MDCs may increase susceptibility to obesity, type 2 diabetes, steatotic liver disease, and other metabolic disorders [3]. Toxicant-associated steatotic liver disease (TASLD, formerly toxicant-associated fatty liver disease or TAFLD [5]) is the new name for the steatotic liver disease that is associated with exposures to chemical pollutants. While carbamate insecticide exposures have been associated with liver and blood lipid disorders, hepatic steatosis is more well-described for organochlorine, organophosphorus, and pyrethroid insecticides [6]. However, exposures to the n-methyl carbamate insecticide, bendiocarb, were associated with hepatic steatosis, apoptosis, and perisinusoidal fibrosis in rabbits [7]. Likewise, carbofuran increased blood and liver lipids or free fatty acids in *Clarias batrachia* fish [8]. Unpublished preliminary data from our group demonstrated a possible association between plasma methomyl levels and steatosis severity in NAFLD patients (n = 150) [9]. This preliminary result was somewhat surprising because methomyl is a restricted-use pesticide with no registered residential uses. There are no data in the literature on the potential impact of methomyl exposures on intermediary metabolism including blood and liver lipid disorders in vivo. However, methomyl exposures in adult rats were associated with hepatotoxicity and oxidative stress [10]. Here, we take a reverse translational approach and evaluate the metabolic impact of methomyl exposures in mice. We hypothesize that methomyl exposure will disrupt xenobiotic and intermediary metabolism and promote hepatic steatosis in mice.

## 2. Materials and Methods

Animals and Diets: No human participants were enrolled in this study; thus, the Institutional Review Board (IRB) Statement is waived. Animal use protocols and procedures were approved by the University of Louisville Institutional Animal Care and Use Committee (IACUC #18220; approved May 2020). Forty male, ten-week-old C57BL/6J mice were purchased from Jackson Laboratory (Bar Harbor, ME, USA). This mouse strain was selected as the ‘gold standard’ for metabolic toxicology model systems. This inbred strain is robust and stable to ensure that individual mice are comparable between different experimental groups. Furthermore, we wanted to ensure that we can reproduce and compare our results to similar studies that have investigated methomyl exposures. Additionally, this study only utilizes male mice to act as a baseline to be comparable to previous research. While sex differences undoubtedly exist in toxicological exposures, we wanted to perform this dose response experiment and compare our observations to previous research, which predominately used male mice. All mice were housed in a temperature- and light-controlled room (twelve-hour light-dark cycle) for the duration of study. Mice acclimatized for one week and were fed the standard, autoclaved laboratory rodent chow diet containing 28.7%, 13.1%, and 58.2% kcal/g of protein, fat, and carbohydrates, respectively (5010; LabDiet, St. Louis, MO, USA). Mice were provided ad libitum access to food and water during the study period.

Study and Exposure Paradigm: Methomyl was purchased from AccuStandard (catalog: P-032N; New Haven, CT). The methomyl stock solution was prepared in nanopure water and used daily for 18 consecutive days. The dose of methomyl was selected based on previous studies to elicit toxic effects while avoiding overt toxicity and lethality [11,12,13,14]. Rodent cages were divided into four groups: control (0 mg/kg methomyl), 1 mg/kg methomyl exposure, 2.5 mg/kg methomyl exposure, and 5 mg/kg methomyl exposure. The lethal dose (LD_50_) of methomyl has been estimated to be approximately 20 mg/kg in previous rodent studies, and the current study, at the highest dose, is four times less than this value. Mice were weighed daily and orally gavaged with water control or methomyl-water solutions depending on body weight. Because daily gavage can be stressful to the mice and non-palatable, in order to avoid resistance, the gavage needle was dipped in a sucrose solution to prevent complications and improve animal welfare [15]. Glucose tolerance testing was performed at day 14 of methomyl exposure. Following our exposure paradigm, research animals were euthanized, and tissue collection was performed on day 21. Blood collection from the inferior vena cava was performed followed by euthanasia via exsanguination. One mouse experienced mortality several days prior to planned euthanasia, likely due to a gavage-related injury.

Liver Histology: Liver tissues were fixed in 10% neutral buffered formalin for 48 h and then placed in 75% ethanol until tissue processing. Leica Biosystem’s Histocore Autocut Automated Rotary Microtome (Leica Biosystem; Deer Park, IL, USA) was used for tissue sectioning at 5 μm. Tissue sections were stained with hematoxylin (catalog: 72,804; Richard Allen; Kalamazoo, MI, USA) and eosin (catalog: HT110316-500 mL; Sigma Aldrich; St. Louis, MO, USA) (H&E) to assess hepatic morphology. Liver sections were also stained with periodic acid (catalog: P7875-25 g) and Schiff reagent (catalog: 3952016-500 mL) (PAS) to determine glycogen content (Sigma Aldrich; St. Louis, MO, USA). H&E and PAS images were captured with Cell Sense Software on an Olympus BX43 microscope at 10-, 20-, and 40-times magnification (Tokyo, Japan). Tissues were also embedded in optimal cutting temperature (OCT) compound (Tissue-Tek; Torrance, CA, USA), and 10 μm sections were cut using a HM525Nx Cryostat (Thermo Scientific; Walldorf, Germany). Frozen liver sections were stained with Oil Red O purchased from Sigma Aldrich (catalog: 26,079) to assess lipid accumulation. The Oil Red O protocol performed was modified from the IHC World Oil Red O Staining Protocol (IHC World, Woodstock, MD, USA). Quantification for Oil Red O stain was performed using ImageJ (v1.53k) software (National Institute of Health; Bethesda, MD, USA) by analyzing 5 random fields of view at 20-times magnification of 5 random livers in each group and the percent area of positive Oil Red O staining averaged depending on grouping. Finally, BODIPY 493/503 fluorescent stain was performed to stain for neutral lipids with a counterstain, DAPI, for nuclei on hepatic frozen sections. Stained tissue sections were fixed in 4% paraformaldehyde and mounted with FluorSave reagent (345789-20 mL, Millipore; Burlington, MA, USA). BODIPY immunofluorescent stained images were captured using 20×, 40×, and 60× magnification with an ECHO Revolve microscope (A BICO Company, San Diego, CA, USA).

Glutathione Analysis: Redox potential measurements were made as described in a previous manuscript [16]. Liver tissue was removed, weighed, and homogenized in 20 volumes of 5% perchloric acid, 0.2 M boric acid and 10 μM γ-glutamylglutamate. After centrifugation to pellet precipitated proteins, the supernatant was derivatized with iodoacetate and dansyl chloride to produce S-carboxymethyl, N-dansyl derivatives that were analyzed by HPLC (Waters Corporation, Milford, MA, USA) to determine molar concentrations of cysteine, cystine, glutathione, and glutathione disulfide. The Nernst equation was then used to determine the plasma redox potential according to the formula: Eh = E0 + 30 × log([oxidized]/[reduced]2), where E0 = −250 mV for Cys/CySS and −264 mV for GSH/GSSG. This method assumes 1 mg of liver tissue protein corresponds to 5 uL of intracellular volume.

Blood Chemistry Analysis: Liver enzymes, triglycerides, glucose, cholesterol, and lipoproteins (HDL, LDL, VLDL) were measured with Lipid Panel Plus diskettes (catalog:400-0030; Abaxis Inc.; Union City, CA, USA) on the Piccolo Xpress Chemistry Analyzer (Abbott; Abbott Park, Chicago, IL, USA). Analysis for each assessment was performed according to the manufacturer’s protocol.

Hepatic Triglyceride and Cholesterol Measurement: Hepatic triglycerides and cholesterol were extracted based on established protocols [17]. Triglyceride and cholesterol standards were used to generate a standard curve to quantify extracted lipids (catalog: T7531-STD, C7509-STD; Point Scientific; Canton, MI, USA). Extracted lipids were colorimetrically measured with a microplate absorbance reader with a wavelength of 500 nm (BioTek Gen 5; Winsooki, VT, USA). Cholesterol and triglycerides were then expressed as mg/g of liver tissue.

qRT-PCR Analysis: RNA-STAT60 was used to homogenize liver tissues and total RNA was isolated (catalog: CS-502; Tel-test Inc.; Friendswood, TX, USA). RNA quantity and purity was assessed with a Nanodrop OneC spectrometer (Thermo Scientific) (catalog: 701-058112; Madison, MI, USA). RNA was reverse transcribed to yield cDNA using single step cDNA synthesis reagent, QScript (catalog: 95048-500; Quantabio; Beverly, MA, USA). A CFX384™ Real-Time System (Bio-Rad; Hercules, CA, USA) was used to perform qRT-PCR. iTaq Universal Probe Supermix from Bio-Rad was used during qRT-PCR set up (catalog: 1725134; Hercules, CA, USA). Relative mRNA expression was calculated based on the 2^−ΔΔCt^ method [18]. Glyceraldehyde-3-Phosphate Dehydrogenase (*Gapdh*) was used as the housekeeping gene (catalog: 4351309; Applied Biosystems; Waltham, MA, USA). Fold induction was calculated and normalized to the control group, 0 mg/kg methomyl, which was normalized to 1. All genes of interest (TaqMan probes) were purchased from ThermoFisher Scientific (Waltham, MA, USA) and are cataloged in Supplemental Table S1.

Statistical Analysis: One-way analysis of variance (ANOVA) was used where different methomyl exposure doses were compared. GraphPad Prism (v9.2) for Mac (GraphPad Software Inc.; La Jolla, CA, USA). The alpha level of 0.05 (*p* ≤ 0.05) was used to determine significance. Significance is designated by the asterisk character ‘*’. One asterisk indicates significance below 0.05, where four asterisks indicate significance below 0.0001. Grubb’s test was used to determine significant outliers in each dataset.

## 3. Results

### 3.1. Methomyl Exposure Altered Body Composition and Glucose Tolerance

**Figure 1:**
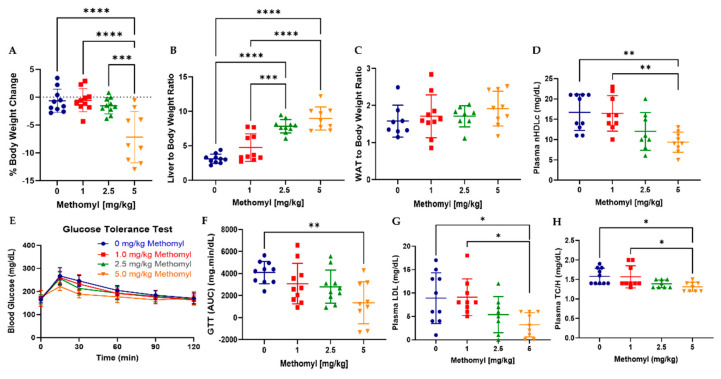
Metabolic disruptions caused by chronic methomyl exposure in mice. (**A**) Percent body weight changes. (**B**,**C**) Liver- and WAT-to-body-weight ratios, respectively. (**D**) Plasma non-HDL cholesterol. (**E**) Glucose tolerance test. (**F**) respective area under the curve. (**G**) LDL cholesterol, respectively. (**H**) Total cholesterol to HDL ratio. Data are reported as mean ± SEM. * *p* < 0.05, ** *p* < 0.01, *** *p* < 0.001, and **** *p* < 0.0001. Abbreviations: AUC, area under the curve; GTT, glucose tolerance test; min, minutes.

First, a comparison of percent body weight change (Figure 1A) between experimental groups revealed that mice exposed to 5 mg/kg methomyl lost significantly more weight (28 ± 18% body weight) than mice treated with vehicle control or lower methomyl doses (~2–6% body weight) despite similar levels of food consumption (Supplemental Figure S1). Methomyl dose-dependently increased the liver-to-body-weight ratio from approximately 4% to 8% body weight at the highest dose (Figure 1B). Meanwhile, the white-adipose-tissue (WAT)-to-body-weight ratio was unchanged by all methomyl doses (Figure 1C). The testes-to-body-weight ratio was increased from 0.75% to 0.85% by all methomyl doses tested relative to control mice (Supplemental Figure S2).

Regarding intermediary metabolism, methomyl 5 mg/kg had a significantly lower glucose tolerance test area under curve (GTT AUC) than vehicle control (*p* = 0.0035) (Figure 1E,F). However, a statistically insignificant trend towards reduced GTT AUC was observed for the lower doses in a stepwise fashion. Fasting plasma glucose was significantly decreased by methomyl 5 mg/kg ~35% (*p* = 0.0153) (Supplemental Figure S3). Plasma non-HDL cholesterol (nHDLc, Figure 1D), LDL cholesterol (Figure 1G), and total cholesterol-to-HDL ratio (TC/H, Figure 1H) were significantly reduced by methomyl 5 mg/kg vs. vehicle control or methomyl 1 mg/kg. Thus, methomyl exposure was associated with a dose-dependent increase in the liver to body weight ratio, as well as decreased body weight and improved glucose tolerance and blood lipids at the highest dose tested.

### 3.2. Methomyl Exposure Altered Hepatic Xenobiotic and Intermediary Metabolism but Did Not Cause Liver Injury

**Figure 2:**
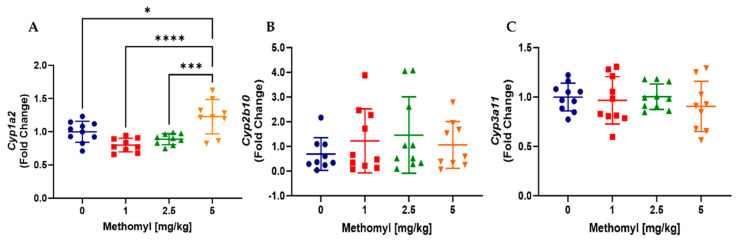
Effects of methomyl exposure mRNA expression of hepatic cytochrome P450 enzymes. (**A**) Cyp1a2. (**B**) Cyp2b10. (**C**) Cyp3a11. Data are reported as mean ± SEM. * *p* < 0.05, *** *p* < 0.001, **** *p* < 0.0001.

Given the role of the liver in pesticide detoxification, we next analyzed whether methomyl exposure altered mRNA expression of selected cytochrome P450 enzymes (Figure 2A,B). Cyp1a2 expression was increased approximately 1.3-fold by methomyl at 5 mg/kg, but Cyp2b10 and Cyp3a11 expression were unchanged. This is consistent with activation of the aryl hydrocarbon receptor (AhR) but not the constitutive androstane receptor (CAR) or the pregnane X receptor (PXR).

**Figure 3:**
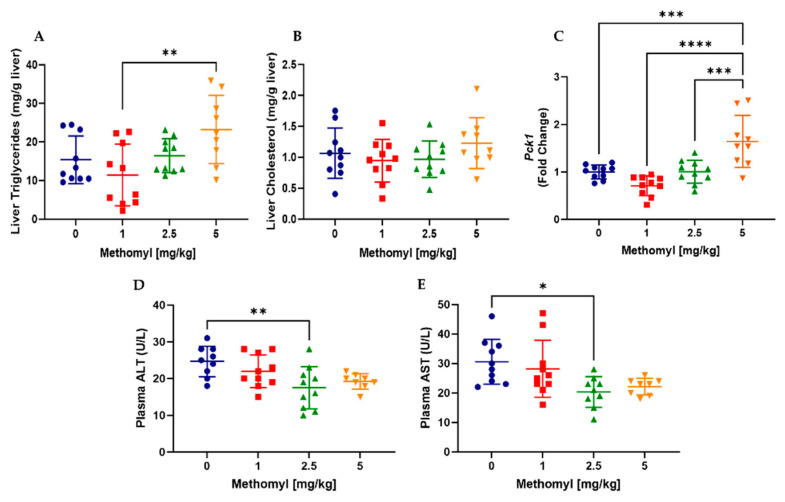
Effects of methomyl exposure on biomarkers of hepatic intermediary metabolism. (**A**) Liver triglycerides. (**B**) Liver cholesterol. (**C**) Liver Pck1 mRNA expression. (**D**,**E**) Plasma ALT and AST activity. Data are reported as mean ± SEM. * *p* < 0.05, ** *p* < 0.01, *** *p* < 0.001, **** *p* < 0.0001. Abbreviations: ALT, alanine aminotransferase; AST, aspartate aminotransferase; Pck1, phosphoenolpyruvate carboxykinase.

Next, biomarkers of hepatic intermediary metabolism were evaluated. Quantified liver triglycerides were significantly increased (x2) by methomyl exposure 5 mg/kg vs. 1 mg/kg, with a trend vs. vehicle control (*p* = 0.089) (Figure 3A). Hepatic cholesterol levels were unchanged by all methomyl doses (Figure 3B). Consistent with the observed relative fasting hypoglycemia with 5 mg/kg methomyl (Supplemental Figure S3), mRNA expression of phosphoenolpyruvate carboxykinase (Pck1), the rate limiting step in hepatic gluconeogenesis, was significantly increased 1.7-fold (*p* = 0.0006) (Figure 3C). Despite the observed alterations in hepatic xenobiotic and intermediary metabolism, plasma alanine aminotransferase (ALT) and aspartate aminotransferase (AST) were unchanged (Figure 3D,E).

### 3.3. Histological Evaluation of Liver Steatosis and Glycogen Content

**Figure 4:**
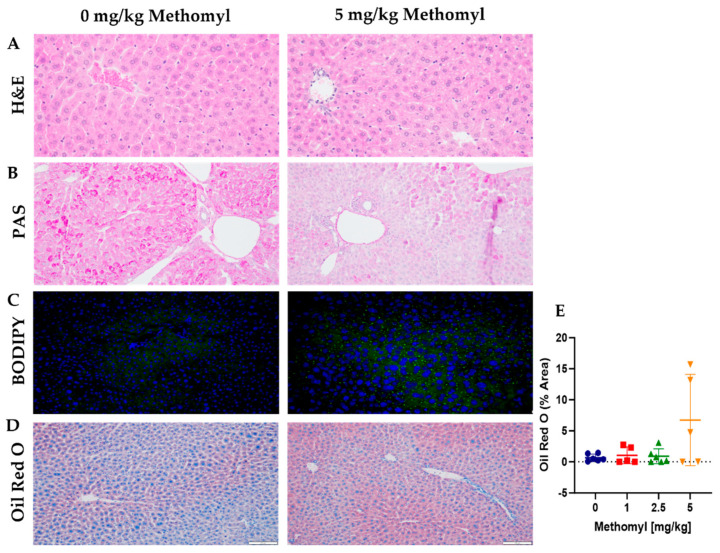
Characterization of hepatic lipids and glycogen by immunohistochemistry and immunofluorescence. (**A**) Liver H&E staining. (**B**) Liver PAS staining. (**C**) Liver BOPIDY immunofluorescence. (**D**) Liver Oil Red O staining. (**E**) Quantitation. Scale bars represent 100 µm. Data are reported as mean ± SEM. Labels represent *p* value. Abbreviations: H&E, Hematoxylin and Eosin; PAS, Periodic Acid Schiff; BODIPY, Boron-Dipyrromethene.

Based on the increase in liver triglycerides, we aimed to characterize the degree of liver steatosis by hematoxylin and eosin (H&E), Oil Red O, and boron dipyrromethene (BODIPY) fluorescent staining. H&E staining revealed increased vacuolization in mice exposed to 5 mg/kg methomyl (Figure 4A). Lobular inflammation and hepatocyte ballooning were not apparent. Increased neutral lipids by BOPIDY immunofluorescence and Oil Red O staining (Figure 4C–E) were observed at the highest methomyl dose consistent with hepatic steatosis. By contrast, liver glycogen content was qualitatively observed to be decreased by methomyl as seen with the periodic acid–Schiff (PAS) stain in Figure 4B.

### 3.4. Effects of Methomyl Exposure on Liver Oxidative Stress

**Figure 5:**
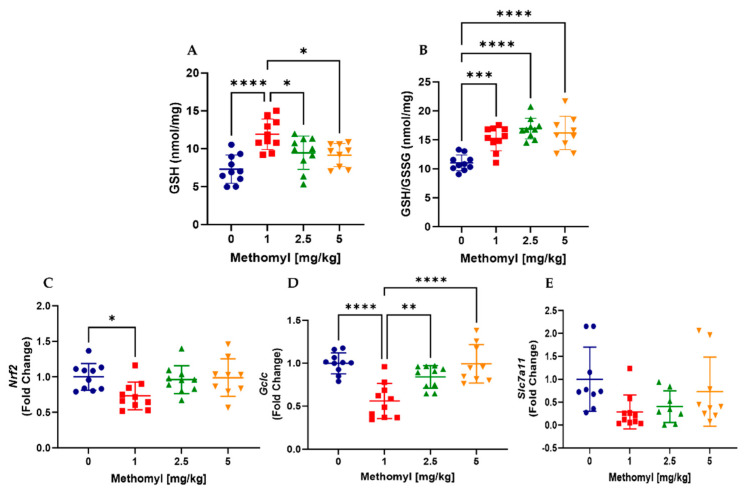
Effects of methomyl exposure on liver oxidative stress. (**A**,**B**) Liver GSH and GSH to GSSG ratio, as measured by HPLC. (**C**–**E**) Liver mRNA levels of *Nrf2*, *Gclc*, and *Slc7a11*, respectively. Data are reported as mean ± SEM. * *p* < 0.05, ** *p* < 0.01, *** *p* < 0.001, and **** *p* < 0.0001. Abbreviations: GSH, Glutathione (reduced); GSSG, Glutathione (oxidized); *Nrf2*, Nuclear Factor, Erythroid Derived 2, like 2; *Gclc*, Glutamate-Cysteine Ligase, Catalytic subunit; *Slc7a11*, Solute Carrier Family 7 Member 11.

Reduced (GSH) and oxidized (GSSG) glutathione were measured in liver and the ratio determined. Hepatic GSH was increased ~5 nmol/mg vs. vehicle control only at the lowest methomyl dose tested (Figure 5A). In contrast, the GSH:GSSG ratio was increased at all doses of methomyl exposure vs. vehicle control (Figure 5B). Multiple genes contribute to liver glutathione production, including the transcription factor, nuclear factor 2-related factor 2 (*Nrf2*) and its target genes, glutamate-cysteine ligase catalytic subunit (*Gclc*), and solute carrier family 7 member 11 (*Slc7a11*). Methomyl (1 mg/kg) significantly reduced mRNA expression (vs. control) of *Nrf2* by approximately 30% (Figure 5C) and *Gclc* by approximately 40% (Figure 5D), with a non-significant trend towards reduced *Slc7a11* expression (*p* = 0.0506, Figure 5E).

## 4. Discussion

In the current study, we characterized the liver and systemic metabolic effects of the n-methyl carbamate insecticide, methomyl. Methomyl was administered by gavage to mimic human exposures association with food contamination by pesticides. In support of the hypothesis, methomyl exposures disrupted xenobiotic and intermediary metabolism and promoted hepatic steatosis in mice. Many of methomyl’s observed effects were either limited to the highest dose (5 mg/kg) or greatest at that dose, which was also shown to induce *Cyp1a2* mRNA expression consistent with AhR activation. Importantly, other pollutants that activate the AhR, such as 2,3,7,8-tetrachlorodibenzo-p-dioxin or PCB126, have previously been shown to exert similar metabolic effects as those observed here for methomyl, including hepatic steatosis and decreased body weights [19,20]. However, methomyl increased the testes:body weight ratio at all doses tested, implicating additional mechanisms. Based on the overall results, methomyl appears to be an endocrine and metabolism disrupting chemical with liver effects including TASLD.

Methomyl exposures resulted in a dose-dependent decrease in body weight independent of food consumption, with a significant weight loss occurring in the 5 mg/kg exposure group. This weight loss is consistent with a previous report in female mice exposed to methomyl (5 mg/kg) for 90 days [21]. The study also documented decreased ovarian follicles and female infertility with methomyl exposure. Methomyl was previously shown to cause reproductive toxicity in male rats as measured by decreased fertility index and testosterone levels as well as increased testicular damage [22]. Previous experiments have noted decrease in testes weight when exposed to methomyl [22,23]. Our finding of an increased testes:body weight ratio requires confirmation in subsequent studies. Although methomyl decreased body weight, the white-adipose-tissue-to-body-weight ratio was unchanged. Increased urination, a possible sign of cholinesterase inhibitor toxicity, was qualitatively observed in some methomyl treated mice. Future studies should perform body composition via imaging to rigorously evaluate fat and lean body mass over time and utilize metabolic cages to measure urine output and perform calorimetry.

Methomyl exposures were associated with alterations in liver and blood lipid alterations. Key findings included increased hepatic steatosis and decreased plasma LDL with methomyl 5 mg/kg exposures. Despite the observed increase in hepatic steatosis, methomyl did not increase liver oxidative stress, injury, or inflammation. First, we observed that at the termination of the study, liver-to-body-weight ratios were increased in a dose-dependent manner. Other n-methyl carbamate studies using either methomyl or bendiocarb also reported increased hepatic vacuolization or steatosis [7,12], corroborating our findings. Increased vacuolization is typically an indication of acute hepatocellular injury where hepatocytes swell in morphology. This could be a marker of methomyl’s acetylcholinesterase inhibitory effects which induces respiratory distress, limiting the amount of oxygen available to cells [24]. While n-methyl carbamate exposures were previously associated with increased hepatic apoptosis and perisinusoidal fibrosis, that study tested a different carbamate in a different species and with a different exposure protocol [7]. In contrast to the increased blood lipids associated with carbofuran exposures in fish [8], here, we reported decreased plasma LDL in methomyl-exposed mice. Consistent with our results, methomyl exposures were previously associated with histologic liver toxicity without transaminase elevation in mice [11]. While that study reported increased liver lipid peroxidation with reduced glutathione, we found an increased GSH:GSSH ratio. These differences could be explained by the different mouse strains and exposure protocols used. Most importantly, the last methomyl dose administered in our protocol was given 3 days before tissue collection. This is a potential limitation of our study because it allows time for exposure-associated oxidative stress to resolve and antioxidant levels to rebound through compensatory mechanisms such as *Nrf2* activation. Indeed, other studies also point to a pro-oxidant role for methomyl in rats [13] and mice [25].

In addition to disruption of lipid metabolism, methomyl also impacted carbohydrate metabolism. Specifically, methomyl exposures decreased fasting blood glucose, the GTT AUC, and hepatic glycogen, while increasing the mRNA expression of the gluconeogenesis gene, phosphoenolpyruvate carboxykinase. The liver is the primary organ where glycogenolysis and gluconeogenesis occurs [26]. These processes play important roles in maintaining blood glucose levels between meals when glucose is not readily available as an energy source [27]. While data are limited for n-methyl carbamates, organophosphate exposure activated glycogen phosphorylase, leading to glycogen breakdown and, subsequently, decreased liver glycogen [4,28]. In our study, the gene (*Pck1*) encoding for an essential enzyme of gluconeogenesis, phosphoenolpyruvate carboxykinase, was significantly increased in the highest dose methomyl group. Hepatic gluconeogenesis may have been upregulated to compensate for the observed glycogen depletion in the setting of relative hypoglycemia.

While this study is insightful with respect to methomyl’s metabolic toxicity, we must report its limitations. First, this study did not incorporate female mice in any of our methomyl exposure groups. Certainly, sexual dimorphic responses are prevalent in metabolism disruption, and methomyl is no exception. Previous works have illustrated that females may have a more robust glutathione response in handling oxidative species [29]. It could be possible that there may be no significant change in GSH or GSSG levels in females and less vacuolization in hepatocytes. Additionally, one methomyl study using similar exposure doses as our study highlighted a significantly higher increase in hepatic injury biomarkers in females compared to males [30]. Further studies are required to understand sexual dimorphic responses to methomyl toxicity. While this study utilized a daily exposure method, methomyl was only administered acutely. The use of methomyl, as an insecticide, likely occurs at lower doses for extended periods of time. For instance, exposures during agricultural use could occur over several weeks to months at a time. Finally, the last methomyl dose was administered on day 18, but mice were euthanized, and tissues were collected on day 21. This temporal separation could adjust some of the acute glutathione and gene expression data (*Nrf2*) where these levels may have begun to fall back to basal conditions. However, this was implemented to observe lingering toxicity after three days of methomyl cessation.

## 5. Conclusions

In conclusion, we observed that methomyl led to numerous mixed effects on global metabolism, liver lipid and energy homeostasis, and liver injury and oxidative stress. Methomyl is a metabolism-disrupting chemical with numerous negative health effects across the body. For instance, altered organ weights, lipid and carbohydrate metabolism, and glutathione metabolism indicate significant underlying toxicity. Future mechanistic studies are required in order to understand how methomyl induces these changes to cause or exacerbate liver disease. The potential mechanisms and impact of n-methyl carbamate insecticides such as methomyl on metabolic health and diseases, including toxicant-associated steatotic liver disease (TASLD), warrant further investigation.

## Data Availability

Not applicable.

## Supplementary Materials

The following supporting information can be downloaded at: https://www.mdpi.com/article/10.3390/metabo13080901/s1, Supplemental Figures S1–S3: Food consumption, testes to body weight ratio, and plasma glucose levels; Table S1: List of genes used for qRT-PCR analyses.
