# Supplementary material for: Investigating the Acute Metabolic Effects of the N-Methyl Carbamate Insecticide, Methomyl, on Mouse Liver
Source: Metabolites. 2023 Aug 1;13(8):901. doi: 10.3390/metabo13080901 (PMC10456691; doi:10.3390/metabo13080901)
Supplement: Supplementary file 1 [file metabolites-13-00901-s001.zip › metabolites-2545183-supplementary.pdf]

Investigating the Acute Metabolic Effects of the N-Methyl Carbamate Insecticide, Methomyl, on Mouse Liver

Supplemental Materials

Amy M. Groswald[1], Tyler C. Gripshover[2], Walter H. Watson[1, 3-5], Banrida Wahlang[1-6], Jianzhu Luo[1,3], Loretta L. Jophlin[1,3-5], Matthew C. Cave[1-8]

1 Division of Gastroenterology, Hepatology, and Nutrition, Department of Medicine, School of Medicine, University of Louisville, Louisville, KY 40202, USA
2 Department of Pharmacology & Toxicology, School of Medicine, University of Louisville, Louisville, KY 40202, USA
3 The Hepatobiology and Toxicology Center, University of Louisville, Louisville, KY 40202, USA
4 The University of Louisville Alcohol Research Center, Louisville, KY 40202, USA
5 The Center for Integrative Environmental Health Sciences, Louisville, KY 40202, USA
6 The University of Louisville Superfund Research Center, Louisville, KY 40202, USA
7 Department of Biochemistry & Molecular Genetics, School of Medicine, University of Louisville, Louisville, KY 40202, USA
8 Robley Rex Veterans Affairs Medical Center, Louisville, KY, USA.

Supplemental Table S1. List of housekeeping gene and Taqman probes of interest for study.

Taqman probe names	Abbreviated name	Assay ID
Glyceraldehyde-3-phosphate Dehydrogenase (Purchased separately from Applied Biosystems)	*Gapdh*	Ref: 4351309
Nuclear Factor, Erythroid Derived 2, like 2	*Nrf2*	Mm00477784
Glutamate-Cysteine Ligase, Catalytic subunit	*Gclc*	Mm00802658
Solute Carrier Family 7 Member 11	*Slc7a11*	Mm00442530
Phosphoenolpyruvate Carboxykinase 1	*Pck1*	Mm01247058
Cytochrome P450 family 1, subfamily a, member 2	*Cyp1a2*	Mm00487224
Cytochrome P450 family 2, subfamily b, polypeptide 10	*Cyp2b10*	Mm01972453
Cytochrome P450 family 3, subfamily a, polypeptide 11	*Cyp3a11*	Mm00731567

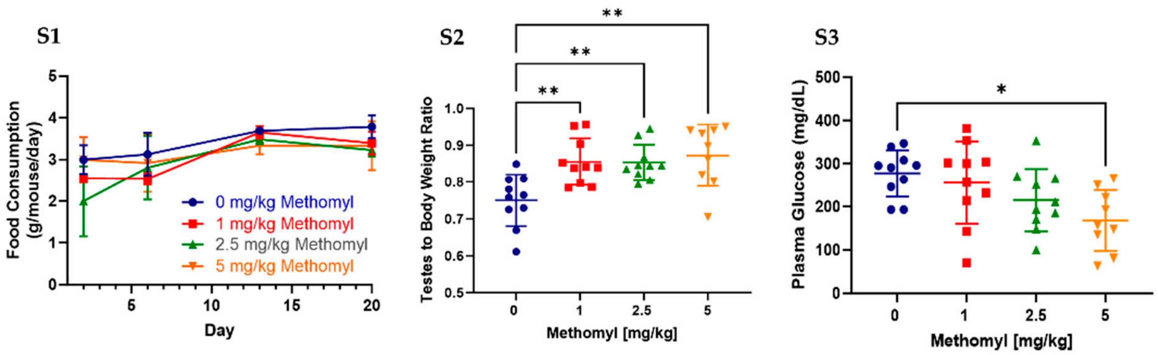

Supplemental Figures S1-S3. Food consumption was similar between all groups **(S1).** Testes to body weight ratio was increased by all Methomyl doses tested. **(S2).** Plasma glucose was decreased by Methomyl (5 mg/kg) **(S3)**. *$p<0.05$, **$p<0.01$.
